# Synthesis and characterization of silver nanoparticles from (bis)alkylamine silver carboxylate precursors

**DOI:** 10.1007/s11051-017-3827-5

**Published:** 2017-03-23

**Authors:** Pawel Uznanski, Joanna Zakrzewska, Frederic Favier, Slawomir Kazmierski, Ewa Bryszewska

**Affiliations:** 1 Centre of Molecular and Macromolecular Studies, PAS, Sienkiewicza 112, 90-363 Lodz, Poland; 2 ICGM - UMR5253- Equipe AIME, Université Montpellier II, 2 Place Eugène Bataillon - CC 1502, 34095 CEDEX 5 Montpellier, France

**Keywords:** Silver carboxylate, Amine-silver carboxylate adducts, Nanoparticles, Silver, Synthesis method

## Abstract

A comparative study of amine and silver carboxylate adducts [R_1_COOAg-2(R_2_NH_2_)] (R_1_ = 1, 7, 11; R_2_ = 8, 12) as a key intermediate in NPs synthesis is carried out via differential scanning calorimetry, solid-state FT-infrared spectroscopy, ^13^C CP MAS NMR, powder X-ray diffraction and X-ray photoelectron spectroscopy, and various solution NMR spectroscopies (^1^H and ^13^C NMR, pulsed field gradient spin-echo NMR, and ROESY). It is proposed that carboxyl moieties in the presence of amine ligands are bound to silver ions via chelating bidentate type of coordination as opposed to bridging bidentate coordination of pure silver carboxylates resulting from the formation of dimeric units. All complexes are packed as lamellar bilayer structures. Silver carboxylate/amine complexes show one first-order melting transition. The evidence presented in this study shows that phase behavior of monovalent metal carboxylates are controlled, mainly, by head group bonding. In solution, insoluble silver salt is stabilized by amine molecules which exist in dynamic equilibrium. Using (bis)amine-silver carboxylate complex as precursor, silver nanoparticles were fabricated. During high-temperature thermolysis, the (bis)amine-carboxylate adduct decomposes to produce silver nanoparticles of small size. NPs are stabilized by strongly interacting carboxylate and trace amounts of amine derived from the silver precursor interacting with carboxylic acid. A corresponding aliphatic amide obtained from silver precursor at high-temperature reaction conditions is not taking part in the stabilization. Combining NMR techniques with FTIR, it was possible to follow an original stabilization mechanism.

**Graphical abstract:**
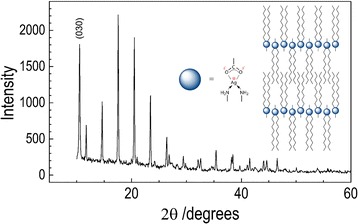
The synthesis of a series (bis)alkylamine silver(I) carboxylate complexes in nonpolar solvents were carried out and fully characterized both in the solid and solution. Carboxyl moieties in the presence of amine ligands are bound to silver ions via chelating bidentate type of coordination. The complexes form layered structures which thermally decompose forming nanoparticles stabilized only by aliphatic carboxylates.

The synthesis of a series (bis)alkylamine silver(I) carboxylate complexes in nonpolar solvents were carried out and fully characterized both in the solid and solution. Carboxyl moieties in the presence of amine ligands are bound to silver ions via chelating bidentate type of coordination. The complexes form layered structures which thermally decompose forming nanoparticles stabilized only by aliphatic carboxylates.

## Introduction

Aliphatic carboxylate salts of silver (C_n_H_2n+1_COOAg) are well-known precursors for preparation of silver colloids (Abe et al. 1998; Wang et al. 1999). Many papers report the preparation of silver nanoparticles (NPs) by decomposition of silver carboxylates in solution (Wu et al. 2006) or in melt (Bokhonov et al. 2014; Keum et al. 2008). Silver NPs (García-Barrasa et al. 2011) along with gold NPs (Prasad et al. 2008; Zhou et al. 2015) and, in less extent, with cooper NPs (Bunge et al. 2003) attract researchers’ attention not only due to its shape and size-dependent plasmonic properties but also as effective antibacterial agents (Furuzono et al. 2013) and metallic inks (Dong et al. 2009; Kim 2013; Vo et al. 2010) applied for preparation of conductive paths used in printing of low-cost electronic circuits for flexible electronics (Wu et al. 2006). The synthesis of silver nanoparticles (Ag NPs) is well described in the literature (García-Barrasa et al. 2011) and concerns thermolysis (Kashiwagi et al. 2006; Keum et al. 2008; Yamamoto and Nakamoto 2003) and reduction with commonly reducing agents such as acrylic or ascorbic acid (Vo et al. 2010), hydrazine (N_2_H_4_) (Dong et al. 2009) or sodium borohydride (NaBH_4_) in biphasic medium (Sarkar et al. 2005), hydrogenolysis (Uznanski and Bryszewska 2010), sonolysis (Veith et al. 2012), etc. Thus, thermolysis of carboxylate metal complex with no use of solvent, stabilizer, or reducing agent should be conducted at high temperatures (~250 °C) since too low temperature leads to a stabilization shell composed of silver ions rather than the fully reduced silver atoms (Shim et al. 2008; Szczęsny and Szłyk 2013). Other methods require the use of solvent, stabilizer, or reducing agent and thus the post-reaction cleaning procedures connected with removing by-products (Bromberg et al. 2010; Rao and Trivedi 2005).

A unique method of preparing Ag NPs was presented by Nakamoto et al., where homologous silver(I) carboxylates and tertiary aliphatic amines in mild reaction conditions result in a good control of silver nanoparticles over the size and distribution (Yamamoto et al. 2006). NPs were capped by carboxylate groups derived from precursors, while the surface of the silver core was free of amine. A large amount of excessive tertiary amine forms a convenient reaction environment for the reduction of postulated (bis)amine-silver carboxylate intermediate at low temperature (80 °C). However, the intermediate was confirmed for primary amine and silver myristate as 2:1 adduct (Yamamoto and Nakamoto 2003). Thus, the silver nanoparticles can be produced in mild thermal conditions via thermal decomposition of the formed amine adduct. Nano-sized silver particles, based on thermal decomposition reaction of silver carboxylates due to their uniform size, form easily 2D and 3D self-assembled ordered structures (Pileni 2011) called supercrystals which are of great importance in modern analytical methods (Bokhonov et al. 2014).

Alternatively, we have shown recently a direct, high yield method for preparing narrow-sized silver nanoparticles by decomposition of silver carboxylate precursor under H_2_ pressure (3 bar) in a nonpolar solvent at a temperature ~150 °C (Uznanski and Bryszewska 2010). It corresponds to the thermal decomposition of carboxylic acid silver salts at 250 °C, but is faster, reproducible, versatile, and easy to control. The reduction of aliphatic carboxylic acid silver salt in the presence of molecular hydrogen leads to the synthesis of Ag NPs to form the corresponding carboxylic acid:

1 $$ 2{\mathrm{C}}_{\mathrm{n}}{\mathrm{H}}_{2\mathrm{n}+1}{\mathrm{C}\mathrm{OO}}^{-}{\mathrm{Ag}}^{+}\overset{\varDelta, {\mathrm{H}}_2\kern0.5em }{\to }{2\mathrm{Ag}}^0+{2\mathrm{C}}_{\mathrm{n}}{\mathrm{H}}_{2\mathrm{n}+1}\mathrm{COOH} $$

while the thermolysis in nitrogen atmosphere is described by a more complicated mechanism:

2 $$ 2{\mathrm{C}}_{\mathrm{n}}{\mathrm{H}}_{2\mathrm{n}+1}{\mathrm{C}\mathrm{OO}}^{-}{\mathrm{Ag}}^{+}\ \overset{\varDelta, {\mathrm{N}}_2\kern1em }{\to }\ {2\mathrm{Ag}}^0+{\mathrm{C}}_{\mathrm{n}}{\mathrm{H}}_{2\mathrm{n}+1}\mathrm{COOH}+{\mathrm{C}}_{\mathrm{n}}{\mathrm{H}}_{2\mathrm{n}}+\mathrm{C}{\mathrm{O}}_2\uparrow $$

A similar reaction in the presence of a primary aliphatic amine also leads to the formation of silver nanoparticles and by-products in the absence of free molecules of carboxylic acid and amine. The preliminary observation pointed to the crucial importance of the composition of the silver precursor and the role of amine in the decomposition and stabilization mechanism of the formed silver particles. The aim of the present study is to investigate the coordination mode of silver carboxylate in the presence of amine ligands and to elucidate the reaction route of the synthesis of Ag NPs from a series of primary (bis)amine (*n*-octylamine, *n*-dodecylamine)/silver carboxylate precursors (acetate, octanoate, and laureate). In this report, we will explain the role of amine ligands during the synthesis and enlighten their very minor role (absence) in stabilization of the final silver nanocrystals. For studying NP ligands and analyzing their properties, we used various methods which include thermal techniques, infrared spectroscopy, solid-state and solution nuclear magnetic resonance spectroscopy (NMR), XRD powder diffraction, and X-ray photoelectron spectroscopy (XPS).

## Experimental section

### Chemicals

Octanoic acid (99%) (OctAc), lauric acid (99%) (LAc), acetic acid (AcAc), 1-octylamine (99%) (OA), 1-dodecylamine (99%) (DDA), silver nitrate (99%), silver acetate (99%) (AgOAc), and sodium hydroxide (NaOH) were purchased from Aldrich or Alfa Aesar and used as received. Solvents (acetone, cyclohexane, and methanol) were distilled prior to use; water was triply distilled. Silver laureate (AgL) was prepared by a one-pot synthesis by adding aqueous solution of NaOH (0.99 equiv. in 2 mL) to the 14-mmol dispersion of lauric acid (2.8 g/20 mL) in hot water (80 °C). The molar amount of NaOH was 1% lower than that of the acid in order to avoid the reaction of excess alkali with AgNO_3_. Then, an aqueous solution of AgNO_3_ (0.56 g in 20 mL of water) was added to the vigorously stirred solution. The resulting silver carboxylate in the form of white precipitate was collected, washed with water (3×), and dried at 50 °C overnight to give white solid in quantitative yield. In a similar way, silver octanoate (AgOct) was prepared.

### Synthesis of [AgL-2DDA] adduct

The adduct was synthesized using ligand insertion by reacting silver dodecanoate (AgL) with 1-dodecylamine in 1:2 stoichiometry. In AgL suspension (0.4 mmol, 0.1228 g) in dry cyclohexane, 0.8 mmol (0.148 g) of DDA dissolved in 5 mL of dry cyclohexane was added. The reaction mixture was heated at 30 °C for 15 min with stirring. On the completion of reaction, a white powder was isolated and dried under vacuum to give the product with quantitative yield. Yield: 181.94 mg (0.332 mmol, 83%), mp. 69.1 °C. Analysis calc. (%) for (C_12_H_25_NH_2_)_2_AgCO_2_(C_11_H_23_): C, 63.79; H, 11.45; N, 4.13; found (%) C, 65.24; H, 13.24; N, 4.55.

Other adducts such as [AgOAc-2DDA], [AgOct-2OA], [LAc-2DDA], [LAc-2OA], [HOAc-2DDA], and [OctAc-2OA] were prepared in a similar way using the same stoichiometric ratio.

**[AgOAc-2OA]**. ^1^H NMR 500 MHz (benzene-d6); *δ*: 0.92 (t 7.2 Hz, 3H, −CH_3_), 1.2–1.36 (m, CH_2_), 1.40 (s), 1.55 (q, 2H), 2.31 (s, 6H, −O_2_CH_3_), 2.74 (t, H), 3.52 (s, b). ^13^C NMR (benzene-d6); *δ*: 14.73, 23.50, 25.47, 27.85, 30.25, 30.47, 32.70, 34.85, 44.68, 177.36.

**[AgOct-2OA]**. ^1^H NMR 500 MHz (benzene-d6); *δ*: 0.927 (m, 9H, −CH_3_), 1.295 (m, 20H), 1.349 (m, 4H), 1.452 (m, 2H), 1.591 (m, 6H), 1.965 (q, 2H, −C_β_H_2_-CH_2_–COOAg), 2.606 (t, 2H, −C_α_H_2_–COOAg), 2.707 (t, 4H, −C_α_H_2_–NH_2_), 3.16 (s, 4H, −NH_2_). ^13^C NMR 125.77 MHz (benzene-d6); *δ*: 14.72 (OA), 14.75 (AgOct), 23.49 (OA), 23.58 (AgOct), 27.79 (OA), 28.31 (AgOct), 30.24 (OA), 30.43 (OA), 30.45 (AgOct), 31.05 (AgOct), 32.71 (OA), 32.87 (AgOct), 34.79 (OA), 38.89 (AgOct), 44.56 (OA), 180.09 (AgOct).

**[AcAc-2OA]**. ^1^H NMR 200 MHz (benzene-d6); *δ*: 0.902 (t, 6H, −CH_3_), 1.220 (m, 20H), 1.506 (m, 4H), 2.215 (s, 3H, CH_3_–COO^−^), 2.645 (t, 4H, −C_α_H_2_-NH_2_), 5.585 (s, 4H, −NH_2_); ^13^C NMR: 14.71, 23.43, 25.66 (CH_3_–COO^−^), 27.55, 30.05, 30.12, 31.86, 32.58, 41.54, 178.88.

**[OctAc-2OA]**. ^1^H NMR 500 MHz (benzene-d6); *δ*: 0.907 (m, 9H, −CH_3_), 1.226 (m, 18H), 1.31 (m, 6H), 1.55 (m, 8H), 1.904 (q, 2H, −C_β_H_2_–CH_2_–COOH), 2.51 (t, 2H, −C_α_H_2_–COOH), 2.64 (t, 4H, −C_α_H_2_–NH_2_), 5.58 (s, 4H, −NH_2_); ^13^C NMR: 14.72 (OA, OctAc), 23.44 (OA), 23.54 (OctAc), 27.57 (OA), 27.75 (OctAc), 30.06 (OA), 30.16 (OA), 30.36 (OctAc), 30.83 (OctAc), 31.94 (OA), 32.6 (OA), 32.78 (OctAc), 39.46 (OctAc), 41.59 (OA), 181.49 (OctAc).

### Synthesis of Ag^0^NPs

In a typical synthesis of silver nanoparticles, (bis)amine silver adduct, either from AgOAc ([AgOAc-2DDA], 0.4 mmol, 0.2160 g) or AgL ([AgL-2DDA], 0.4 mmol, 0.2708 g), was introduced in a 10-mL round-bottom flask connected to a nitrogen vacuum line and purged with three vacuum/nitrogen cycles. The reactor was placed in a heating jacket equipped with a temperature controller and a magnetic stirrer. The silver carboxylate-(bis)amine complex was heated to 180 °C at a rate of 3 °C/min and kept at this temperature for 20 min. The reaction was cooled down to room temperature by immersing the flask in a cold water bath. The color of the melt changed from light yellow to dark brown as the reaction proceeded. The formed particles were washed out from the row solid product using MeOH and gentle bath sonication. The dispersion process was repeated three times. The final sticky precipitate was dried with a nitrogen flow and collected for further analysis. When dissolved into benzene or cyclohexane, it resulted in a stable dark yellow solution. The supernatant was colorless and had no free carboxylic acid or amine derived from started reactant, as will be discussed later. The main component of supernatant was *n*-dodecyldodecanamide (white solid). ^1^H NMR 500 MHz (CDCl_3_) *δ*: 5.49 (b s, 1H), 3.22 (q, *J* = 6.0 Hz, 2H), 2.136 (t, *J* = 7.69 Hz, 2H), 1.603 (q, *J* = 7.28 Hz, 2H), 1.47 (q, *J* = 7.0 Hz, 2H), 1.42–1.19 (m, 34H), 0.95–0.84 (t, *J* = 7.03 Hz, 6H). ^13^C NMR 125.77 MHz (CDCl_3_) *δ*: 173.42, 39.82, 37.27, 32.24, 30.01, 29.98, 29.95, 29.92, 29.89, 29.84, 29.71, 29.67, 29.65, 27.26, 26.2, 23.01, 14.14.

As control sample, Ag NPs were prepared from AgL precursor (0.4 mmol, 0.1228 g) at 250 °C for 10 min (Abe et al. 1998).

### Characterization techniques

FTIR spectra of the studied samples were obtained using a Jasco 6200 FT-IR spectrophotometer in transmission or attenuated total reflectance (ATR) mode by spreading a drop of the sol in cyclohexane on a substrate (Si plate or Ge ATR crystal) and letting it dry. The spectra were obtained by averaging 64 interferograms with resolution of 4 cm^−1^. Extinction UV–Vis spectra of Ag NPs were collected with a resolution of 1 nm in a quartz cell (*d* = 2 mm) in cyclohexane using a HP UV 8543 diode array spectrometer.

Differential scanning calorimetry (DSC) and thermogravimetry (TG) analyses were recorded in the presence of nitrogen on a DSC 2920 (TA Instruments) and TGA 2950 (TA Instruments), respectively. Approximately 3–4 mg of the material was used, and the heating process was recorded at the rate of 10 °C/min. Microanalysis was performed with a Euro-Vector model 3018 instrument.

1D and 2D NMR spectra were recorded using either a Bruker Advance 200 or DRX 500 spectrometer equipped with a 5-mm triple-resonance inverse Z-gradient probe. All diffusion measurements were made by using the stimulated echo pulse sequence with bipolar gradient pulses. The 2D ROESY measurements were performed with a mixing time of 100 ms. Solution NMR samples were prepared in benzene-d6 and were done at 25 °C. The solid-state cross-polarization magic angle spinning NMR measurements were performed on a 400-MHz Bruker Avance III spectrometer and equipped with a MAS probe head and a 4-mm ZrO_2_ rotor. The spectra were recorded with a proton 90° pulse length of 4 ms, contact time of 2 ms, repetition delay of 4 s, and 8 kHz MAS rotation rate.

Scanning electron microscope (SEM) images were obtained using a Hitachi S-4700 FE-SEM operating between 8 and 12 keV. The substrate for SEM was carbon tape (Agar Scientific). Samples were prepared by deposition of colloidal solution on a substrate.

Surface chemical characterization of NPs thin film was conducted using AXIS Ultra photoelectron spectrometer (XPS, Kratos Analytical Ltd.) equipped with monochromatic Al–K-α X-ray source (1486.6 eV). The power of anode was set at 150 W, and the hemispherical electron energy analyzer was operated at pass energy 20 eV for all high-resolution measurements. The sample area subjected to analysis was 300 × 700 μm in size.

Powder X-ray diffraction patterns were collected using a Panalytical X′PERT MPD diffractometer for a 2θ range of 5° to 120° at an angular resolution of 0.05° using Co-Kα (1.7890 Å) radiation.

## Results and discussion

It is well known that in the presence of tertiary amines, silver alkyl carboxylates significantly lower their decomposing temperature and silver NPs can be produced efficiently even at 80 °C (Yamamoto et al. 2006). Postulated intermediate amine-silver carboxylate complex was not observed so far; however, the analogous one was isolated for primary amines. In the following, results from various experimental methods will be presented, allowing the determination of the role of the amine in thermolysis and the Ag NPs formation. AgOAc and AgL or AgOct were selected to show the role of aliphatic chain in high-temperature synthesis in the presence of OA or DDA.

Interaction between Ag carboxylate and amine takes place in solvents (hexane, cyclohexane, and toluene) as well as in the melt. In nonpolar solvents at room temperature (25 °C), almost instant complex formation was observed between insoluble silver salt and amine when a molar ratio [amine]/[Ag carboxylate] of 2 was chosen, wherein the complex remained insoluble. The same ratio has an effect on the efficient complexing with amine and bivalent metal cations (Li et al. 2004) or ion pairing of amine and carboxylic acid in binary mixtures, as has already been observed in the stabilization and organization mechanism of ZnO nanoparticles (Coppel et al. 2012a, b).

### Thermal characterization

**Fig. 1:**
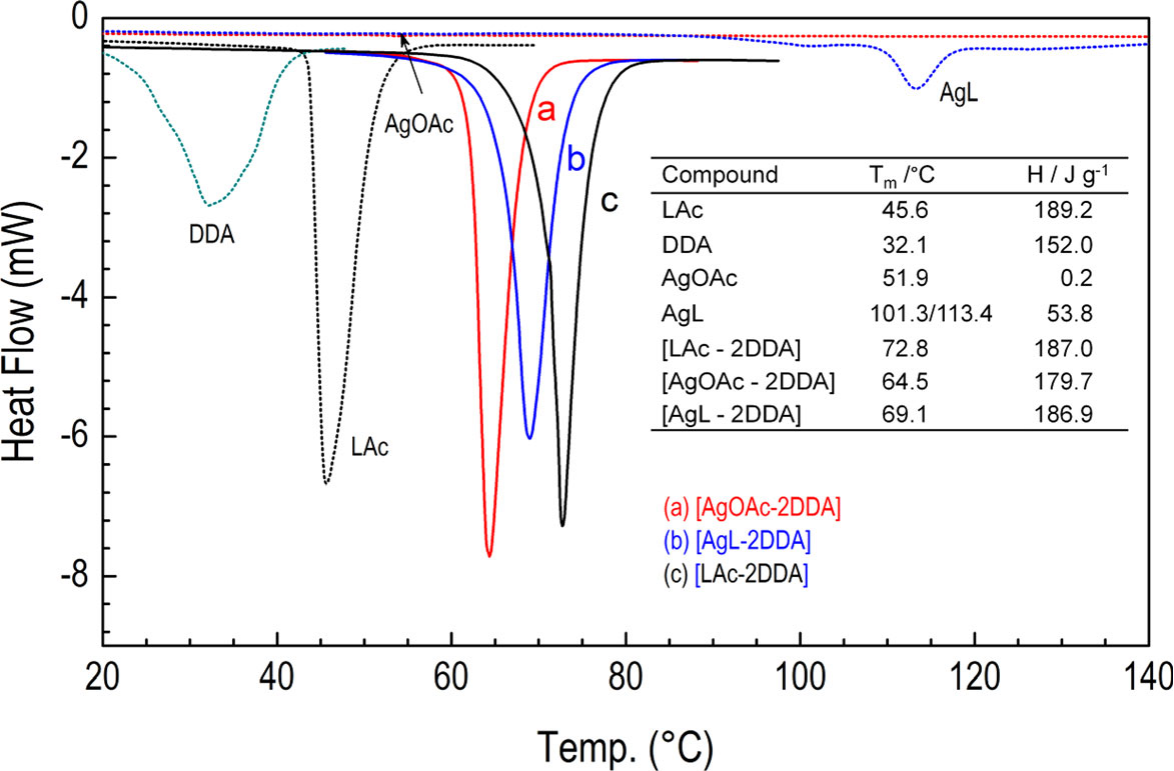
DSC heating scans (10°/min) of (bis)dodecylamine silver carboxylate adducts: (*a*) [AgOAc-2DDA], (*b*) [AgL-2DDA], and (*c*) (bis)dodecylamine carboxylic acid ion-pair [LAc-2DDA]. For comparison traces of pure AgOAc, AgL, LAc, and DDA are also presented. *Inset*: the observed transition temperatures (T_m_) and enthalpy heat (ΔH) for the studied materials

The thermal behavior of the silver carboxylates in the complex with DDA has been explored using a combination of DSC, thermogravimetric analysis (TGA), and mass spectrometry (MS). During thermal scanning (Fig. 1), the samples undergo clear endothermic transformations, temperature position, and enthalpy of which can be related to the composition and the amount of the coordinated ligands. DSC traces of pure dodecylamine, dodecanoic acid, and silver acetate and laureate with the transition temperature T_m_ at 32.1, 45.6, 51.9, and 101.3/111.4 °C, respectively, were substantially modified. In the complexes with amine, these broad peaks show one component sharp endothermic transitions that occur between 60 and 80 °C (Fig. 1 and Table 1). Thus, the melting temperatures are evidently changed compared to those corresponding to pure ligands, especially for AgL which exhibits a series of phase changes at heating above 100 °C. Silver laureate at around 110 °C undergoes a first-phase transition connected with the formation of a new crystalline structure. DSC results suggest a hypothesis that all passivating ligands are coordinated to the silver ion, which in turn imposes the packing of the aliphatic chains and their cooperative arrangements. The well-defined phase transitions occurring upon heating cannot be assigned to pure melting processes of aliphatic chains, and structural transformation of these materials has to be taken into account. The details of this transition are currently being studied using temperature-dependent FTIR and XRD.

**Table 1:** Infrared bands and vibrational mode assignments for silver carboxylates and (bis)amine-silver carboxylate complexes

Peak positions (cm^−1^)				Assignments
AgOAc	AgL	[AgOAc-2DDA]	[AgL-2DDA]	
		3268sh, 3194, 3103, 3049	3335, 3175, 3099	ν_as_(NH_2_), ν_s_(NH_2_), overtone
	2954	2955	2954	ν_as_(CH_3_)
	2917	2915	2918	ν_as_(CH_2_)
	2870	2871	2872	ν_s_(CH_3_)
	2849	2850	2849	ν_s_(CH_2_)
		1620	1608	δ(NH_2_)
1516	1519	1549	1551	ν_as_(COO^−^)
	1471	1471	1470	δ(CH_2_) scissoring
1415sh	1422, 1412sh	1392	1392	ν_s_(COO^−^)
1385				δ(CH_3_)
1345				ν(C-COO^−^)
	1325–1171	1272–1127	1325–1150	ω(CH_2_) progression
1035		1066		ν(CO)
931		910		δ(COO^−^)?
	718	716	718	ρ(CH_2_) rocking

**Fig. 2:**
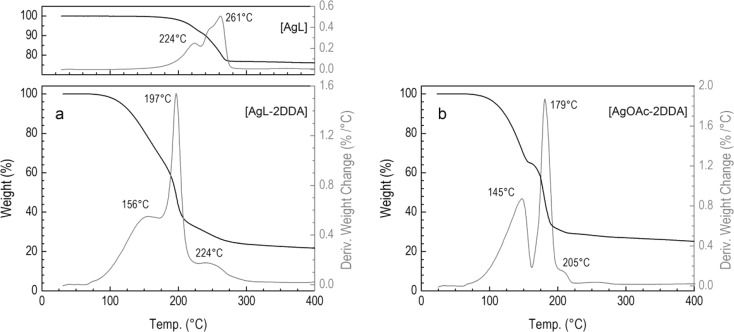
Thermogravimetric traces and their first derivatives (*gray lines*) of **a** [AgL-2DDA] and **b** [AgOAc-2DDA]. Heating rate in the nitrogen atmosphere was 10 °C/min. *Inset* in plot **a** shows thermal decomposition of pure AgL

For both [AgOAc-2DDA] and [AgL-2DDA] complexes, thermogravimetric analysis was also performed under nitrogen atmosphere (Fig. 2) to examine the composition and thermal stability. From the first derivative curve, it follows that for (bis)amine silver laureate adduct, the weight loss is a three-step decomposition with decomposition maximum temperatures at *T* = 156, 197, and 224 °C. The weight loss between 80 and 180 °C can correspond to the loss of DDA molecules weakly coordinated in the complex with silver salt. Between 180 and 280 °C, the weight loss is assigned to the thermal decomposition of the silver laureate. This is supported by the molar ratio of Ag to laureate and dodecylamine estimated from the relative weight loss of inorganic to organic fragments (Ag/laureate/amine = 19.4:33.8:46.8). Pure silver laureate exhibits weight loss between 150 and 300 °C (inset in Fig. 2a). The decomposition events of (bis)amine silver acetate start at lower temperature due to the low decomposition temperature of pure silver acetate and run similarly through a three-step decomposition at around 145, 179, and 205 °C (Fig. 2b). Thus, silver acetate/(bis)amine complex could be well suited for material processing purposes.

**Fig. 3:**
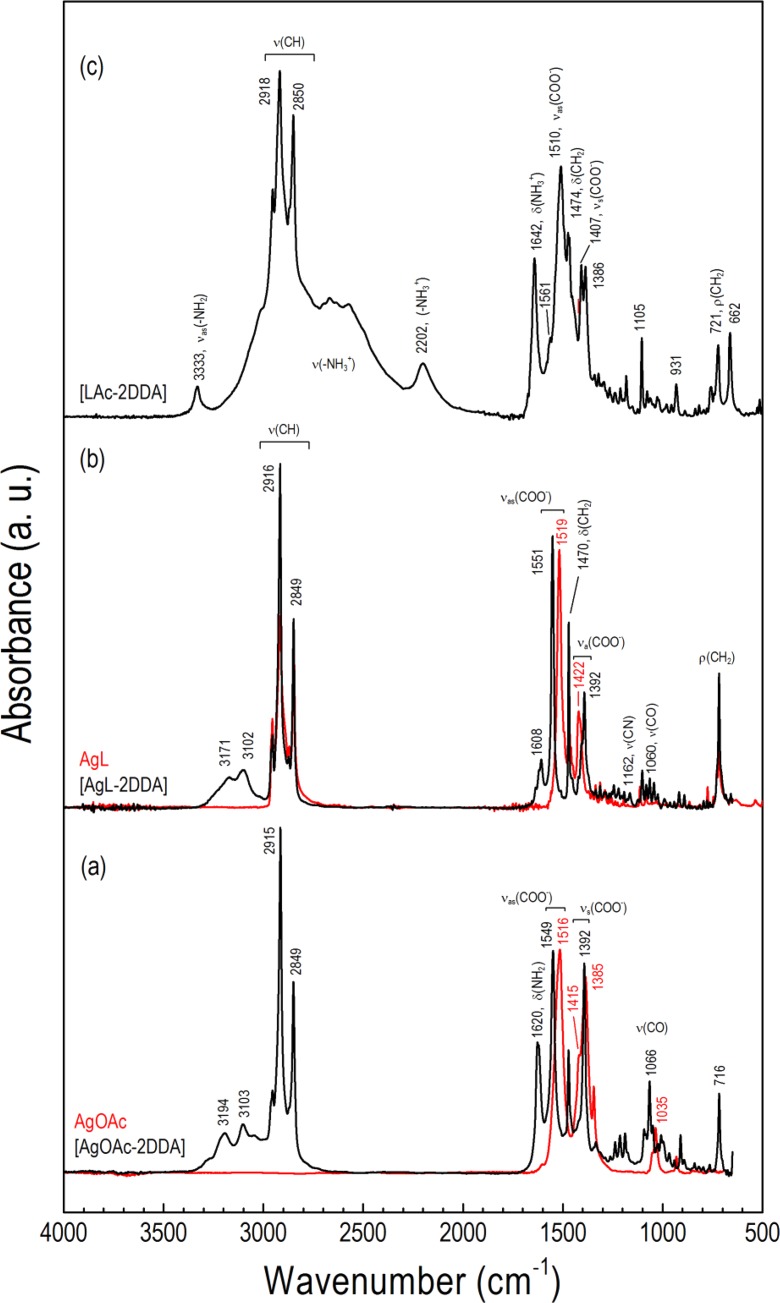
FTIR powder spectra of **a** AgOAc and [AgOAc-2DDA] adduct, **b** AgL and [AgL-2DDA] adduct, and **c** DDA[LAc^−^-DDA^+^] ion pair

Formation of soluble and quite stable complexes of AgOAc and AgL in nonpolar solvents in the presence of dodecylamine at stoichiometric ratio 1:2 was evidenced in FTIR studies (Fig. 3 and Table 1). Before discussing the spectra, it would be informative to analyze analogous complexes formed between LAc and DDA in the mixture of 1 equiv. of both LAc and DDA [LAc-DDA] (ammonium carboxylate salt) and in the mixture of 1 equiv. of LAc and 2 equiv. of DDA [LAc-2DDA] (Fig. 3c). The high-frequency region of infrared spectra for aliphatic LAc, DAA, [LAc^−^-DDA^+^], and DDA[LAc^−^-DDA^+^] mixture is characterized by two strong bands assigned to the antisymmetric ν_as_(CH_2_) and symmetric ν_s_(CH_2_) methylene C–H stretching modes at 2918 and 2850 cm^−1^, respectively, and two weak bands at 2952 and 2871 cm^−1^ assigned to the asymmetric ν_as_(CH_3_) and symmetric ν_s_(CH_3_) stretching modes of the methyl in the alkyl chain, respectively (Lee et al. 2002; Pelletier et al. 2003; Wu et al. 2004). These bands for LAc overlap stretching vibrations of hydroxyl ν(OH) ranging from 3400 to 2200 cm^−1^ and originating from H-bonded dimers. The specified position of CH_2_ stretching peaks indicates a high percentage of all-trans conformations. In DDA spectrum, amine asymmetrical (ν_as_(NH_2_) = 3330 cm^−1^) and symmetrical (ν_s_(NH_2_) = 3253 cm^−1^) N–H stretching modes are also observed. In turn, for ion-paired ammonium carboxylate [LAc^−^-DDA^+^] and its mixture with amine DDA[LAc^−^-DDA^+^], the observed broad band between 3200 and 2100 cm^−1^ originates from the antisymmetric and symmetric N–H stretching of the resultant ammonium anion −NH_3_^+^ of the ion-pair [LAc^−^-DDA^+^] formed in apolar cyclohexane with the well-developed combinational band at 2202 cm^−1^. Moreover, characteristic N–H amine stretching vibrations fade for primary amine salts (Goodreid et al. 2014) though not completely for the [LAc-2DDA] mixture. The infrared low-frequency region of LAc contains bands from the C═O stretching vibration of the carboxylic group at 1699 cm^−1^ in the H-bonded dimer, methylene bending mode δ(CH_2_) at 1468 cm^−1^, and methylene rocking ρ(CH_2_) at 722 cm^−1^. The spectrum also clearly shows several bands at 1299, 1410, and 937 cm^−1^ due to C–O stretching vibration and in-plane and out-of plane bending of the C–OH group, respectively. All bands from the carboxylic acid groups disappear from the spectra of ion-pair [LAc^−^-DDA^+^] and DDA[LAc^−^-DDA^+^]. Instead, new bands at 1510 and 1407 cm^−1^, ascribed to carboxylate asymmetric and symmetric vibrations, respectively, are observable. The amine bond deformations δ(NH_2_) present in dodecylamine solid as composite band at 1606 cm^−1^ are also observed as one intensive band at 1642 cm^−1^ for both [LAc^−^- DDA^+^] and DDA[LAc^−^-DDA^+^] ion pair. This indicates that the ion pair formed in the solution is stable in the solid. Three bands observed for DDA[LAc^−^-DDA^+^] at 3333, 1561, and 1386 cm^−1^, which are not presented in [LAc^−^-DDA^+^] ion pair, are ascribed to DDA in the mixture of amine, ammonium, and amine in interaction with the ion pair.

Similar interactions occur between silver alkanoate and dodecylamine. Figure 3 shows FTIR ATR spectra of AgOAc and AgL and the spectra of their complexes with DDA. The selected vibrational mode assignments and frequencies are collected in Table 1. When 2 equiv. of DDA is added to 1 equiv. of AgL or AgOAc in cyclohexane at room temperature, insoluble silver salt forms (bis)amine silver carboxylate adduct. These complexes are stable in the solid state as was checked by thermal characterization (vide supra), and their presence manifests in the changes of FTIR spectra of both silver carboxylate and amine groups (Fig. 3a, b). For example, peaks for carboxylate asymmetric stretching ν_as_(COO^−^) and symmetric stretching ν_s_(COO^−^) from carboxylate group in AgL appear at 1519 and 1422 cm^−1^, respectively. They replace two bands typically presented in dimeric form of carboxylic acid due to the presence of carbonyl C═O at 1699 cm^−1^ and hydroxyl C–OH group at 1299 cm^−1^. It is worth mentioning here that analogous frequency shift due to electron delocalization is observed for [LAc^−^-DDA^+^] ion pair (Table 2). For silver *n*-alkanoate interacting with amine, the corresponding peaks were found to be respectively in high frequency (1551 cm^−1^) and low frequency (1392 cm^−1^) shifted from the original peak position. Amine stretching and bending vibrations are also low wavenumber shifted with an intensity increase due to complex formation as compared to bulk amine.

**Table 2:** Observed frequency position of the antisymmetric ν_as_(COO^−^) and symmetric ν_s_(COO^−^) stretches for different coordination modes in carboxylates and acetates in the presence of DDA in the neat phases

Species	ν_as_(COO^−^), cm^−1^	ν_s_(COO^−^), cm^−1^	Δν(=ν_as_-ν_s_), cm^−1^	Coordination mode
AcAc	1755/1728/1698 (monomer/dimer)	1296	459/432/402	
LAc	1696 (dimer)	1299	397	
[AcAc-DDA]	1515	1405	110	Bridging bidentate
[AcAc-2DDA]	1515	1405	110	Bridging bidentate
[LAc-DDA]	1511	1405	106	Bridging bidentate
[LAc-2DDA]	1510	1407	103	Bridging bidentate
NaOAc (H_2_O)^a^	1561	1413	148	Ionic (chelating)
NaL (H_2_O)	1557	1422	135	Ionic (chelating)
AgOAc	1516	1415	101	Bridging bidentate (dimer)
AgL	1518	1415	103	Bridging bidentate (dimer)
[AgOAc-2DDA]	1549	1392	157	Chelating (ionic)
[AgL-2DDA]	1551	1392	159	Chelating (ionic)
Ag NPs^b^	1528	1399	129	Chelating

^a^(Nara et al. 1996)

^b^Ag NPs synthesized from [AgL-2DDA] complex as described in Experimental

**Scheme 1:**
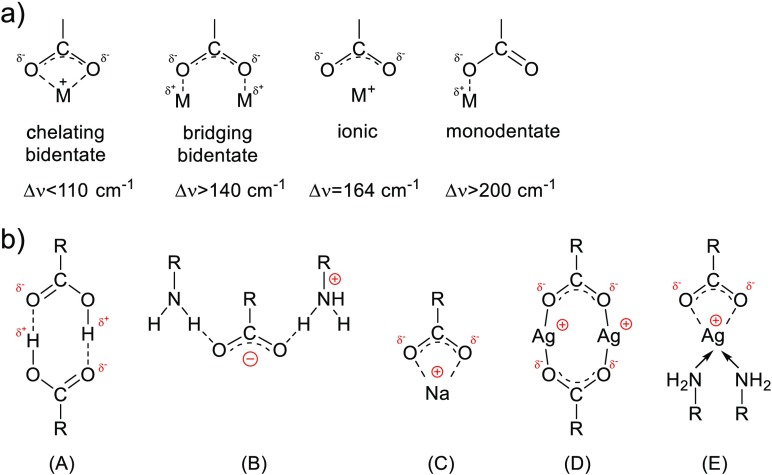
**a** Bonding modes of carboxylate ligand and a metal cation. **b** Proposed bonding modes of carboxylate ligands in: (*A*) carboxylic acid (head-to-head dimer), (*B*) carboxylic acid-(bis)amine ion pair (bridging ionic), (*C*) sodium alkanoate salt (ionic/symmetrical), (*D*) silver alkanoate (bridging bidentate/symmetrical), (*E*) silver alkanoate-(bis)amine adduct (chelating bidentate/symmetrical)

Interaction of amine with silver carboxylate can be considered taking into account changes in the oscillation energy of the carboxyl group (Nelson and Taylor 2014). The type of coordination of carboxylate to metal cations is a consequence of changes in the CO bond lengths and the OCO angle. Thus, it would be useful to discuss the position changes of carboxylate anion COO^−^ stretching frequencies in the presence of amine as the wavenumber separation between COO^−^ antisymmetric and symmetric vibrations Δν(=ν_as_-ν_s_), in a similar way, as was empirically established for the interaction of metal ions with the carboxylate group itself (Gericke and Hühnerfuss 1994; Nara et al. 1996). The frequency difference Δν allows the identification of the type of coordination of the carboxylate group to metal cations (Scheme 1a).

It was found that for bivalent metal cation coordination ability, Δν weakens in the order of monodentate, bridging/ionic bidentate to chelating bidentate form (Ellis et al. 2005; Ohe et al. 1999). The larger band shift for the complex indicates a larger unsymmetrical interaction between the metal ion and the carboxylate group. Monodentate mode of coordination, where only one carbonyl oxygen atom interacts with the metal, due to its lower symmetry than the free ion shows similarities with the spectra of the monomeric form of carboxylic acids. Thus, for monodentate coordination, the asymmetric COO^−^ stretching frequency increases while the symmetric COO^−^ stretch decreases relative to the values observed for free carboxylate ion (Ohe et al. 1999). For monovalent silver carboxylates, an opposite effect is observed with a significant lowering of the asymmetric COO^−^ stretching position and generation of Δν as low as 103 cm^−1^. Based on recent powder XRD studies of the homologous series of metal *n*-alkanoates which revealed that the silver atoms form symmetric dimers in an eight-membered ring (Aret et al. 2006; Tolochko et al. 1998), we propose to correlate the observed stretching separation Δν for AgL with bridging bidentate coordination (Scheme 1). According to previous correlation (Nelson et al. 2015), the frequency difference below 110 cm^−1^ would have suggested a dimeric monodentate coordination mode for silver carboxylates. The opposite effect was observed for (bis)amine-silver carboxylate complex. Due to coordination of two amine ligands to silver ion, carboxylate group is slightly separated and becomes more ionic similarly to highly electropositive sodium carboxylate (Δν = 135 cm^−1^) (Nelson et al. 2015; Wulandari et al. 2015). The larger band shift for the complex with amine suggests chelating character of coordination (Scheme 1). The measured frequencies of the antisymmetric and symmetric COO^−^ stretches for the two silver salts studied, AgL and AgOAc, and for their complexes with amine and silver NPs are shown in Table 2.

The degree of interaction between carboxylic acid and amine measured with the magnitude of splitting Δν between asymmetric and symmetric stretching indicates that the bond order of both CO groups is similar to the silver carboxylate complex; thus, the ion pair can be modeled like two amine molecules interacting symmetrically with two oxygen atoms. Indeed, as can be seen from Table 2 for (bis)amine-carboxylic acid ion pair, the carboxylic stretching frequency at 1696 cm^−1^ (C═O bond) shifts to 1510 cm^−1^ while the C–O vibration is high frequency shifted from 1299 to 1407 cm^−1^. Diminishing the separation frequency between the two bands from Δν = 397 cm^−1^ for LAc to Δν = 103 cm^−1^ for DDA[LAc^−^-DDA^+^], the ion pair can be ascribed to the bridging coordination character of the carboxylic group. As a matter of fact, it is not a strict electrostatic ion pair, rather H-bonded charge-assisted associate (Scheme 1).

### Powder XRD analysis

All X-ray diffractograms of the studied complexes demonstrate a well-developed progression of intense reflections, which can be interpreted in terms of stacked layers. Similar diffraction patterns are observed for layered structures of silver carboxylates (Blanton et al. 2011; Lee et al. 2002) where the closely spaced peaks were attributed to the (*00 l*) indexes. The silver atoms in carboxylate salts are bridged by the carboxylate in the form of dimers in an eight-membered ring, and the dimers are further bonded to each other by longer Ag–O bonds forming, in turn, four-membered rings (Aret et al. 2006; Tolochko et al. 1998). The crystal morphology is led by the stacking of the carbon chains (Aret et al. 2006; Binnemans et al. 2004). The experimental d-interlayer spacing of the silver laureate obtained by determining the average position of the first six measured reflections, (00*2*) to (00*7*), from the small angle region was 34.659 Å. Similar X-ray powder diffractogram was recorded for the 1:2 complex of silver laureate and dodecylamine [AgL-2DDA]. The well-defined reflection peaks forming the d-spacing values are in the ratio 1:½:^1^/_3_...^1^/_6_. Such a diffraction pattern of reflection peaks, as discussed earlier, is consistent with a layered structure. The calculated value of the average d-spacing is 33.490 Å, slightly lower than that for the pure AgL material.

**Fig. 4:**
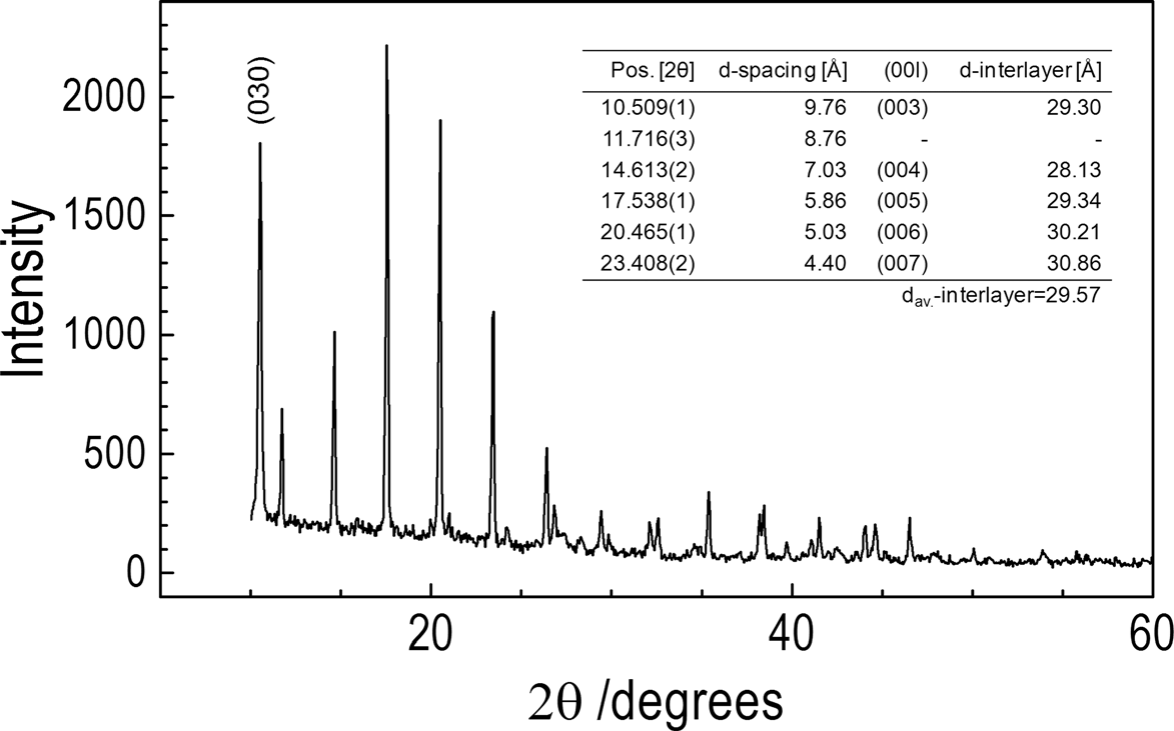
XRD pattern of silver acetate/(bis)dodecylamine. The interlayer spacing calculated from successive (*00 l*) reflections is listed in the inset

Figure 4 shows the XRD patterns of (bis)dodecylamine silver acetate complex. In the inset of Fig. 4, the interlayer distances derived from different reflections are listed. The index of the first diffraction peak assumed to be (*003*) (the (*001*) and (*002*) reflections are outside the detector range) and corresponds to a d-interlayer spacing of ~29.57 Å. The fully extended dodecylamine molecule can be estimated to be about 18 Å long. The values indicate that each layer of [AgOAc-2DDA] is separated from the neighboring layer by less than twice the length of the amine alkyl chain and suggests interpenetrated aliphatic chains in the bilayer.

### NMR spectroscopic studies

#### Solid-state ^13^C NMR

**Fig. 5:**
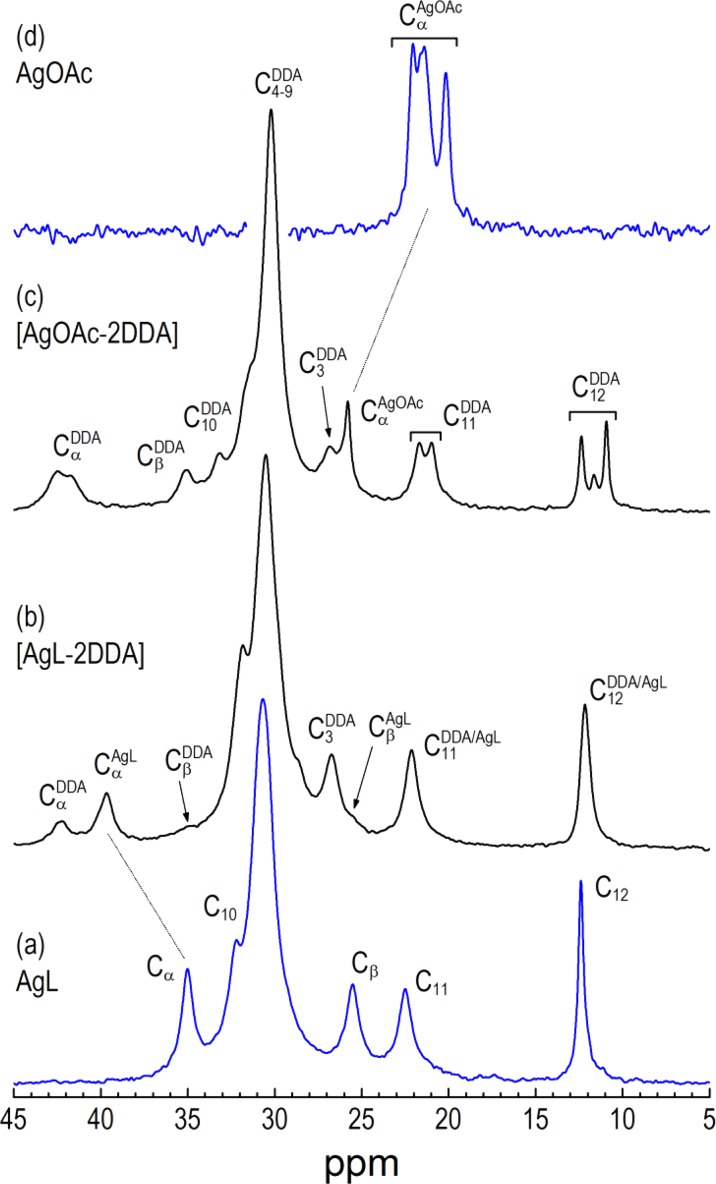
^13^C CP-MAS NMR spectra of the **a** AgL, **b** [AgL-2DDA], **c** [AgOAc-2DDA], and **d** AgOAc complexes. Conditions: contact time: 5 ms; pulse delay: 4 s; spinning rate: 8 kHz

Cross-polarization magic angle spinning (CP-MAS) ^13^C NMR measurements have been performed on the AgOAc, AgL, [AgOAc-2DDA], and [AgL-2DDA] material, and the results are shown in Fig. 5 and Table 3. The chemical shift of the central chain methylenes (30.2 ppm) indicates a high degree of conformational order of aliphatic chains for the three measured samples in accord with the IR data. However, in the case of [AgOAc-2DDA], it was found that the outermost methylene group (C_11_) of the DDA chain must also contain some defects due to resonance splitting and the shift of 21.7 ppm which is below that observed with [AgL-2DDA] powder (22.13 ppm). The methyl group shows also distinguished splitting and high field shift which might suggest mutual interdigitation in the ends of the chains of adjacent layers. The formation of (bis)amine silver carboxylate adduct has a significant effect also on the first two carbon atoms in the alkyl chain (C_α_ and C_β_). We assign the doublet at 42.7/41.77 ppm to C_﻿α﻿_ and the singlet at 35.07 to C_β_ of amine moiety. Similarly, the resonance associated with C_β_ of silver laureate at 35.01 ppm is strongly shifted to 39.66 ppm in the amine silver laureate complex. Thus, the C_β_ of laureate is deshielded in adduct, while carboxyl carbon of the complex is shielded relative to the silver salt.

**Table 3:** The main ^13^C CP-MAS NMR resonance peak position for AgOAc, AgL, [AgOAc-2DDA], and [AgL-2DDA] complexes

	^13^C CP-MAS NMR peak position (ppm)			
	AgOAc	AgL	[AgOAc-2DDA]	[AgL-2DDA]
−COOH	175.32	176.44	174.12	175.80
−CH_3_ (AcAc)	22.04, 21.59, 21.4, 20.16	–	25.78	–
−C_α_ (LAc)		35.0	–	39.66
−C_β_ (LAc)		25.5	–	25.60
−C_11_ (LAc)		22.5	–	22.13
−C_12_ (LAc)		12.40	–	12.16
−C_α_ (DDA)		–	42.47, 41.74	42.20
−C_β_ (DDA)		–	35.06	34.80
−C_11_ (DDA)		–	21.70, 20.98	22.13
−C_12_ (DDA)		–	12.37, 11.64, 10.93	12.16

To understand the nature of complexion between primary amines and silver carboxylates, more data can be gained from solution NMR spectroscopy as changes in ^1^H and ^13^C chemical shifts, relaxation rates, and diffusion coefficients and comparison to one-component solutions of amine and carboxylic acid. The [AgOAc-2DDA] and [AgL-2DDA] adducts of (bis)dodecylamine silver laureate tend to form gels in *d*-benzene at a concentration of 100 mM at 25 °C. Therefore, NMR studies in solution were conducted for octylamine and octanoate complexes and for correspondent acetic/octanoic acid and octylamine as reference mixtures of the studied systems (Coppel et al. 2012a, b; Pagès et al. 2009).

#### Solution NMR spectroscopic studies

**Fig. 6:**
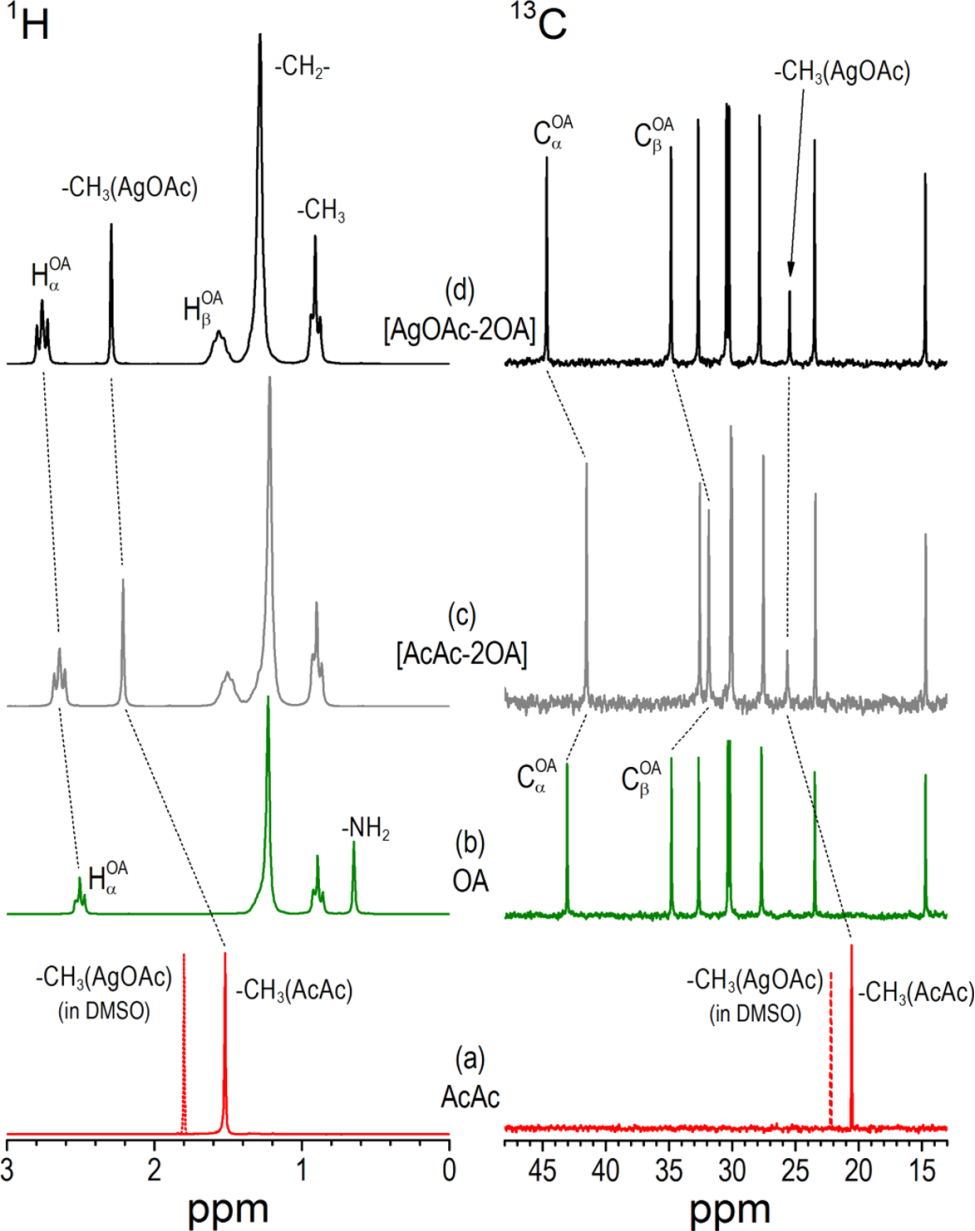
^1^H and ^13^C NMR spectra in *d*-benzene at 298 K of AcAc **a**, OA **b**, 1:1 mixture of AcAc with OA **c**, and 1:2 mixture of AgOAc with OA **d**

**Fig. 7:**
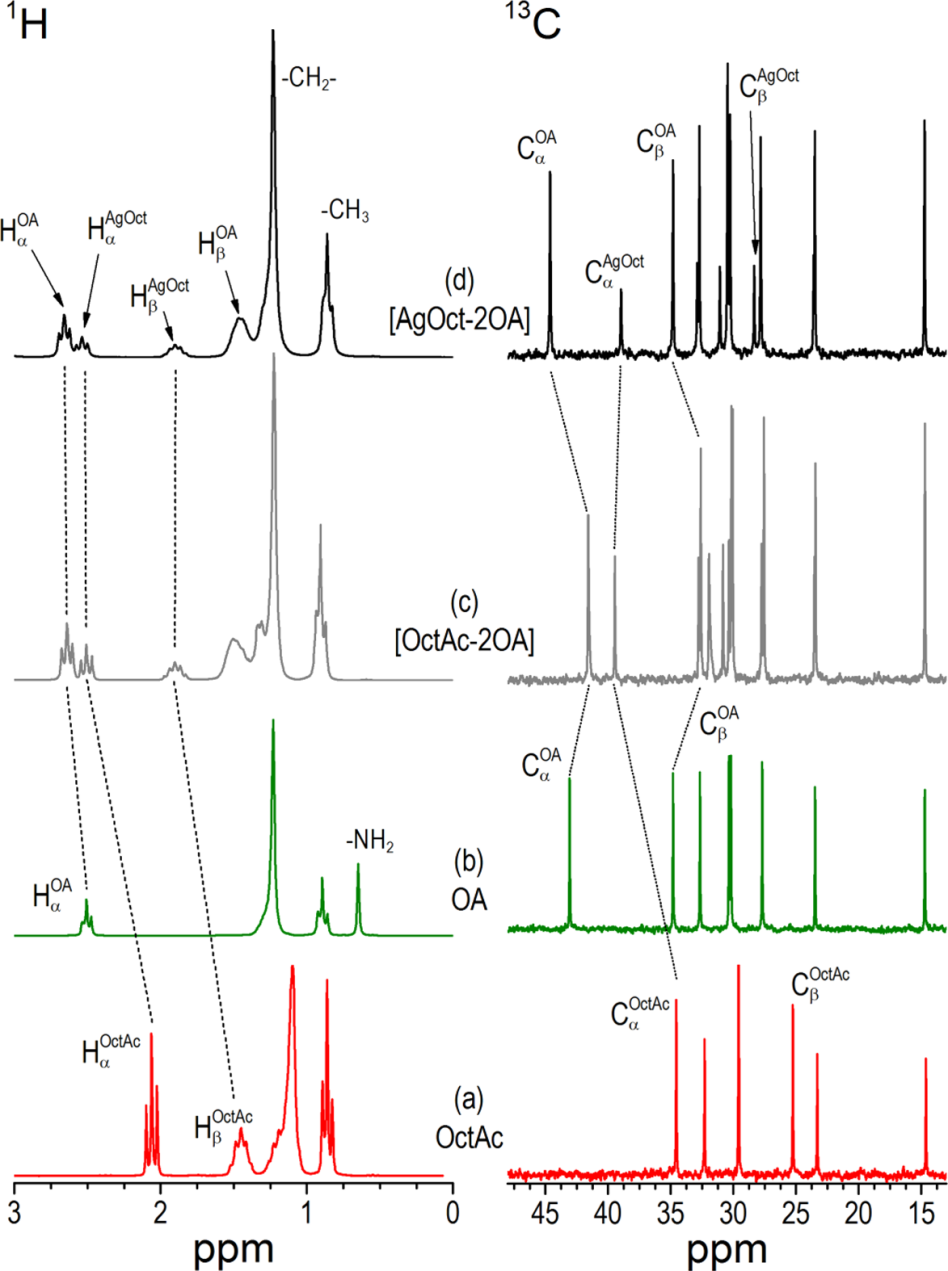
^1^H and ^13^C NMR spectra in *d*-benzene at 298 K of OctAc **a**, OA **b**, 1:1 mixture of OctAc with OA **c**, and 1:2 mixture of AgOct with OA **d**. A concentration of OA was 400 mM

Figures 6 and 7 present ^1^H and ^13^C NMR solution spectra of [AgOAc-2OA] and [AgOct-2OA] complexes together with the spectra of pure OA, AcAc, OctAc, and 1:2 mixtures of [AcAc-2OA] and [OctAc-2OA] as references (Brammer et al. 2001) (silver salts AgOAc and AgOct are not soluble in deuterated benzene). The resonances were assigned using ^1^H spectra, COSY, and ^1^H-^13^C HSQC correlations. For stoichiometric ratios up to at least 4 equiv. of OA and 1 equiv. of Ag carboxylate, only signals from a unique amine and carboxylate species were observed. All ^1^H NMR resonances show analogous shifts when compared to those corresponding to ion-paired ammonium/carboxylate (Fig. 7 traces c and d). On the contrary, ^13^C NMR resonances of the two innermost carbons to the amine and acid groups (C_α_ and C_β_) for amine/silver carboxylate complexes show significant shifts relative to those for carboxylic acid/amine ion pair.

The presence of a single stet of NMR signals may suggest either a stable complex or a complex in a fast exchange with amine. Similar results are obtained for [AgOAc-2OA] system (Fig. 6), with the difference that ^1^H NMR resonances are all deshielded as compared to the CH_3_COOH/amine mixture. For ^13^C spectrum, only the amine C_α_ and C_β_ carbon positions reflect a major difference between ion-paired ammonium/carboxylate system and (bis)amino silver carboxylate complex. For example, the C_α_ carbon of amine is shielded in [OctAc-2OA] ion-pair system as compared to the pure amine, while it is deshielded in [AgOct-2OA] complex. Almost no modification of the proton chemical shifts of the carboxylate group is observed. Therefore, the chemical surrounding of the carboxylate is not affected by the different coordination of ammonium or amine moiety. The chemical shift may result from the screening effect in the presence of silver cations.

Further information on the nature of (bis)amine silver carboxylate adduct was gained from DOSY NMR experiments conducted in pulsed field gradient (PFG). Two main diffusion coefficients for OA and silver carboxylate in 1:2 mixtures can be distinguished in the spectra indicating fast exchange between [RCOOAg-2OA] complexes. Simultaneously, the diffusion coefficients for OA and carboxylate are lower than those measured independently for single-component solutions (Table 4).

**Table 4:** ^1^H and ^13^C chemical shifts (δ in ppm) and diffusion coefficients (D × 10^−10^ in m^2^ s^−1^) of the free octylamine ligand and 1:2 mixtures of octylamine and silver carboxylate at 25 °C in C_6_D_6_. Octanoic acid and acetic acid are listed as references for the corresponding insoluble silver salts

	^1^H and ^13^C NMR peak position (in ppm) and diffusion coefficients (D × 10^−10^ in m^2^ s^−1^)				
	AcAc, OctAc, OA	[AgOAc-2OA]	[AgOct-2OA]	[AcAc-2OA]	[OctAc-2OA]
^1^H					
−CH_3_ (AcAc)	1.52	2.32	–	2.22	–
α-CH_2_ (OctCOO^−^)	2.06	–	2.6		2.51
α-CH_2_ (OA)	2.51	2.66	2.73	2.65	2.64
−CH_3_ (OctCOO^−^)	0.86	–	0.92		0.90
−CH_3_ (OA)	0.90	0.93	0.94	0.90	0.90
^13^C					
−COO^−^(AcAc)	178.46	178.88	–	178.88	–
−CH_3_ (AcAc)	20.57	24.70	–	25.65	–
−COO^−^(OctCOO^−^)	181.45	–	180.10	–	181.49
α-CH_2_ (OctCOO^−^)	34.55	–	38.88	–	39.46
α-CH_2_ (OA)	43.02	44.65	44.56	41.54	41.59
−CH_3_ (OctCOO^−^/OA)	14.63/14.71	14.73	14.75/14.72	14.7	14.72
D					
log D (AcAc^−^)	–	4.3	–		
log D (OctCOO^−^)	11.4	4.3	3.9		
log D (OA)	17.9	10.6	7.2		

**Fig. 8:**
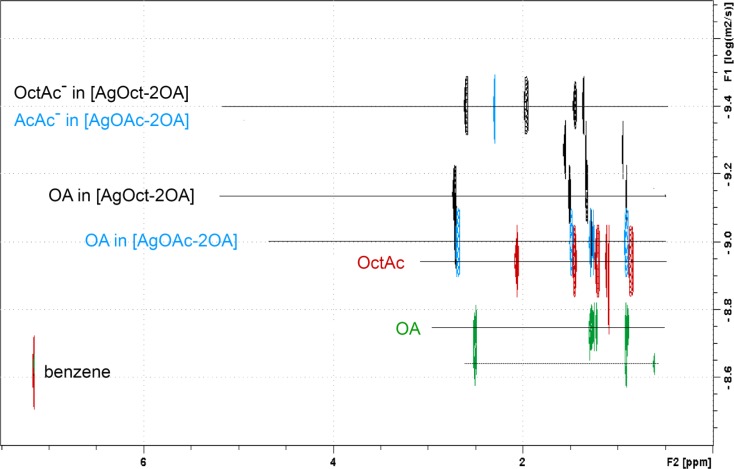
Superposition of the 2D DOSY spectra of OA, OctAc, and (bis)amine silver carboxylate complexes [AgOAc-2OA] and [AgOct-2OA]. All spectra were obtained at 298 K in *d*-benzene

Figure 8 presents the 2D DOSY solution spectra of OA, OctAc, and (bis)amine silver carboxylate complexes, [AgOAc-2OA] and [AgOct-2OA]. Regardless of the system, the diffusion coefficient values for carboxylate and amine are systematically lower than those measured separately for the pure acid or amine in benzene as well as for a model ion-paired [OctAc-2OA] complex. Interestingly, the self-diffusion coefficient of silver octanoate is identical to that of the smaller silver acetate. On the other hand, one can observe two sets of different diffusion coefficients for OA and OctAc^−^ in [AgOct-2OA] adduct and only one set of diffusion coefficients D for OA and AcAc^−^ for the silver acetate adduct [AgOAc-2OA]. Due to the fact that total solubility of silver carboxylates in the presence of amine occurs at 1:2 ratio, one can assume two types of amine ligands, respectively weakly and strongly interacting with Ag carboxylate. The OA in the latter mode of interaction predominates over the first one. This assumption is supported by gel formation of 1:1 mixture of diaminooctane silver octanoate in nonpolar solvents. Twofold excess of aminooctane leaves the spectra unaffected. Still, a single set of peaks is measured for the amine. This suggests weakly interacting amines can exist in fast exchange either with free amines in solution or with strongly (coordinated) ones.

**Fig. 9:**
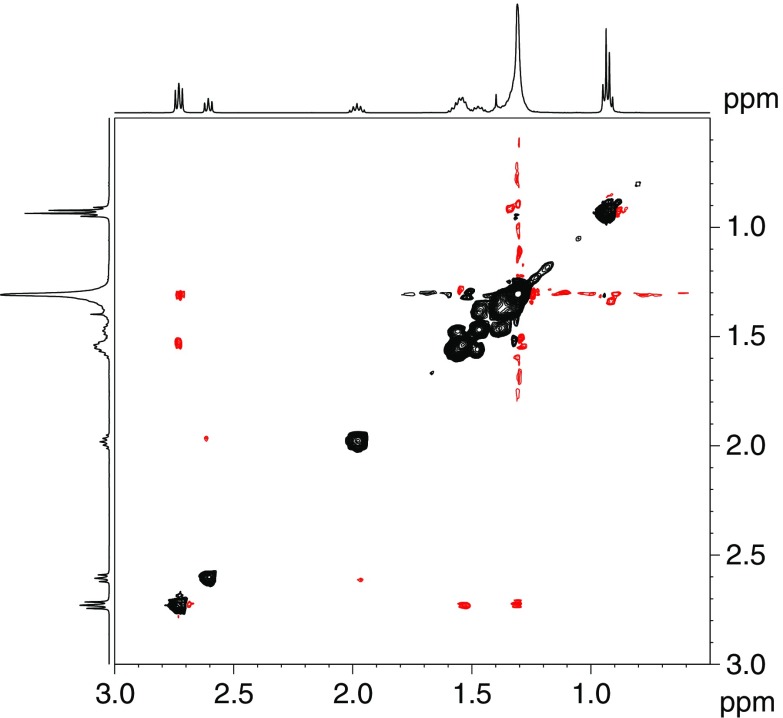
^1^H ROESY experiment for [AgOctAc-2OA] complex in *d*-benzene at 25 °C

The arrangement of (bis)amino-silver carboxylate complex in solution and its dynamic nature can be referred from the NMR ROESY experiment. In the ROESY spectra, α-H_2_ protons of amine and carboxylate are poorly coupled and rather one can easily distinguish intrachain interactions (Fig. 9). This is in contrast to the spectra of octanoic acid in the mixture with 2 equiv. of OA, where off-diagonal cross-peaks of the α protons of OA and OctAc are clearly visible (Pagès et al. 2009).

### Characterization of silver NPs synthesized from bis(amino) Ag carboxylate adduct

Silver NPs were synthesized at 180 °C from [AgL-2DDA] adduct (2 mmol, without solvent) under nitrogen atmosphere. The powder was heated by immersing a Schlenk flask into silicon oil bath and allowed to react for 20 min, during which it gradually turned yellow–brown. The (bis)amine silver carboxylate complex slowly melted and finally afforded homogeneous dispersion containing silver nanoparticles. The reaction was quenched by immersing the flask in a cold water bath. The reaction products are composed of lauric acid/amine, *n*-dodecyldodecanamide, and carboxylate/amine-capped silver NPs. Crude nanoparticles were cleaned and separated by washing out twice with methanol (5 mL) and air dried. Purified dark blue waxy material was easily soluble in organic solvents. The silver fraction for the pure (bis)amine carboxylate sample exactly corresponds to that for nanoparticles obtained by the thermal decomposition under hydrogen pressure (Uznanski and Bryszewska 2010).

**Fig. 10:**
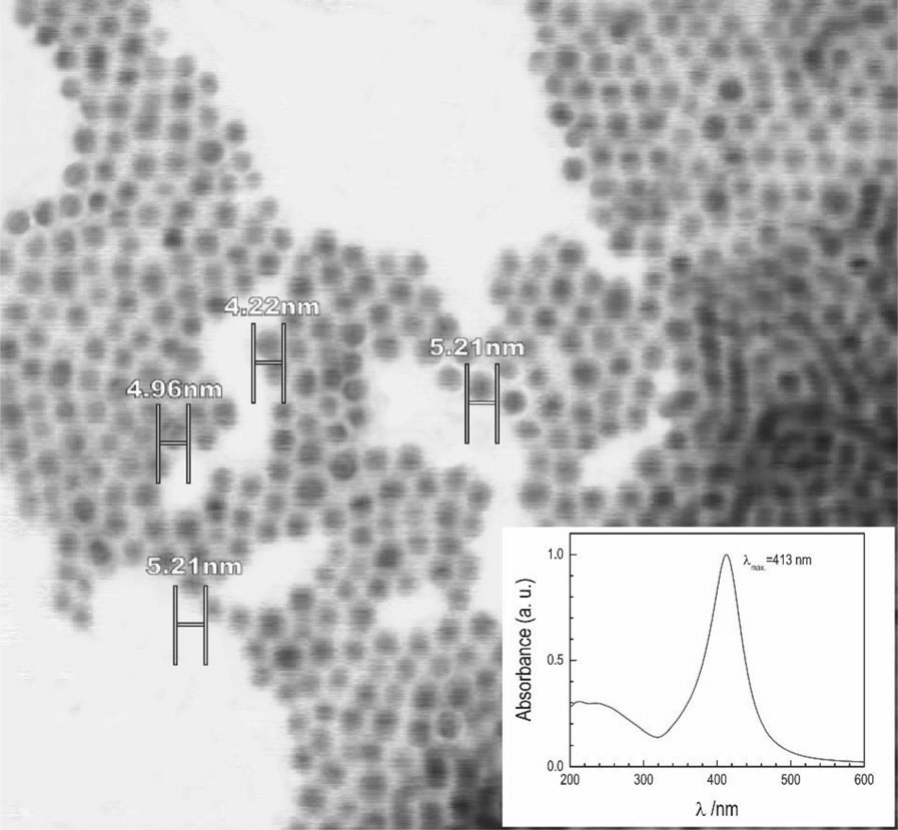
Scanning electron microscope (SEM) image of laureate/amine stabilized silver nanoparticles. The film was drop-casted by putting a drop of cyclohexane-dispersed Ag nanoparticles on carbon tape. *Inset*: UV–Vis absorption spectra of purified Ag NPs from excessive ligands in cyclohexane

Figure 10 shows a SEM image of AgL NPs, the average diameter of which is 5.0 ± 0.2 nm. Their polydispersity, small size, and regular shape are reflected itself in the formation of 2D and 3D supramolecular structures and in almost symmetrical UV–Vis spectrum of plasmon resonance with a maximum at 413 nm (inset in Fig. 10), which is slightly blue shifted as compared to NPs obtained in the presence of tertiary amine (Yamamoto et al. 2006).

**Fig. 11:**
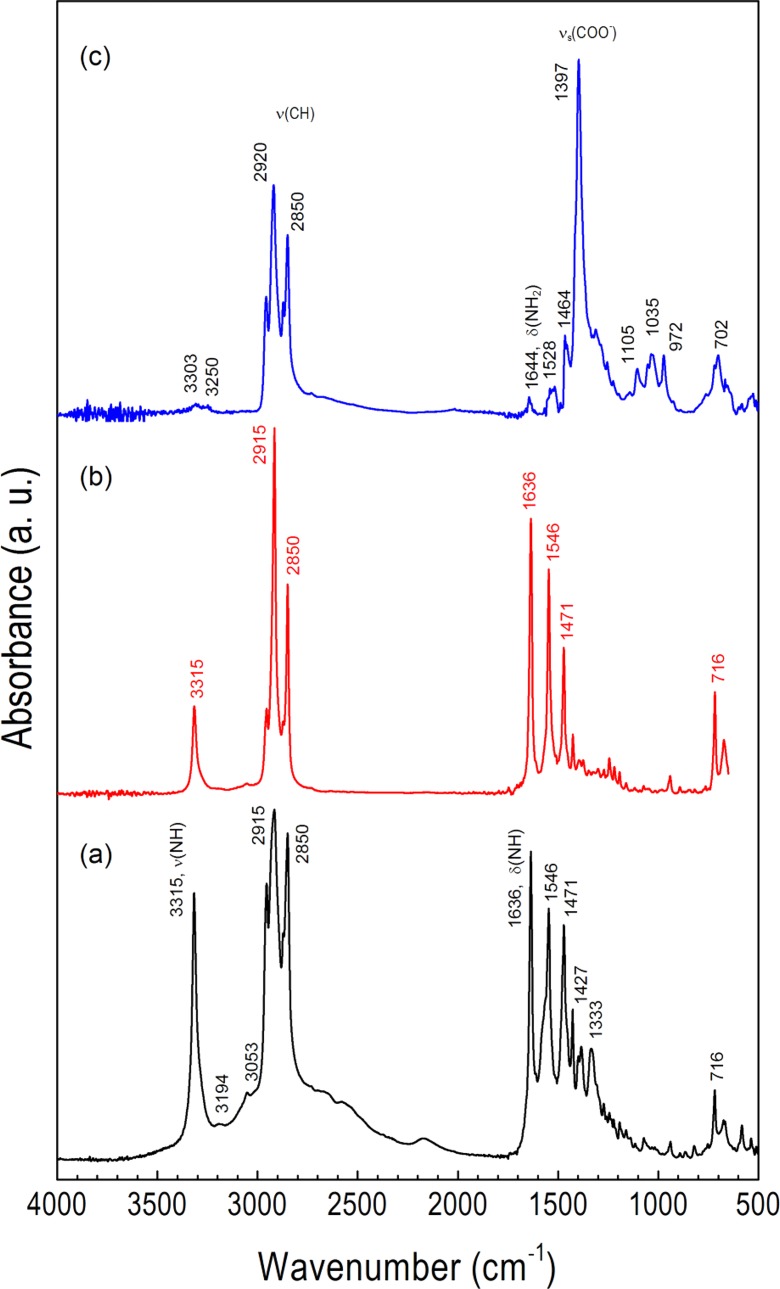
FTIR spectra of **a** the crude products from the synthesis of Ag NPs, **b** the isolated *n*-dodecyldodecanamide by-product, and **c** purified in MeOH Ag NPs

Products from thermal decomposition of [AgL-2DDA] complex are composed of three main components: lauric aid/amine ion pair, *n*-dodecyldodecanamide (Goodreid et al. 2014), and Ag NPs capped with laureate/amine ligands (Fig. 11). As confirmed by FTIR, laureate appears to be the dominant capping agent as it was not replaced by the long chain amine (Fig. 11c). The characteristic vibrational modes for the coordinative carboxylate group when bound to a metal surface are significantly modified as compared to [AgL-2DDA] precursor (Figs. 3b and 11c). The symmetric COO^−^ stretching mode is red-shifted and shows an intense band at 1397 cm^−1^. A low-intensity band at 1528 cm^−1^ is ascribed as a relict vibration of the asymmetrical COO^−^ stretch. Such IR band suppression is often observed at metallic surfaces, and results here from the parallel alignment of the induced dipole moment to the silver surface. Very weak bands at 1644, 3250, and 3303 cm^−1^ are δ(NH_2_) bond deformation and symmetric and asymmetric ν(NH_2_) stretching, respectively, observed for the neat primary amine. The spectrum on Fig. 11b shows very sharp peaks at 3315, 1636, and 1546 cm^−1^ characteristic for amide moiety which was formed during high-temperature synthesis of Ag NPs (Goodreid et al. 2014). Ag NPs from [AgOAc-DDA] are not stable and are not stabilized by acetate and/or amine ligands.

**Fig. 12:**
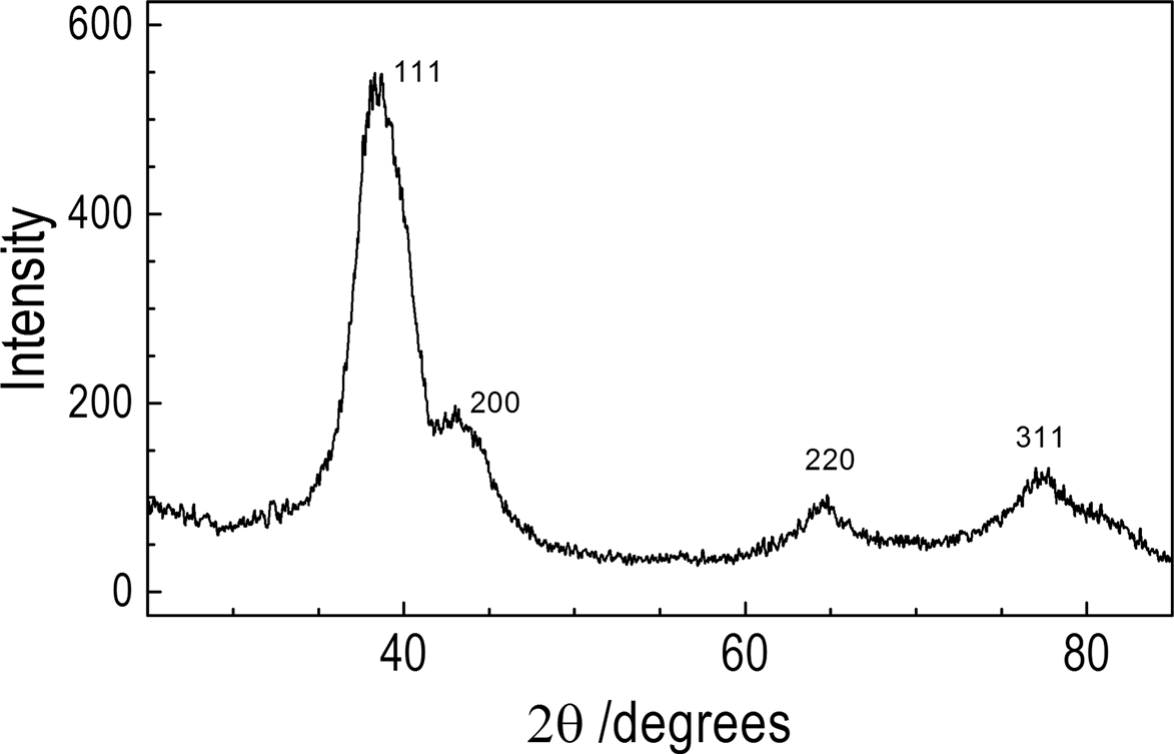
Powder XRD patterns of silver nanoparticles from [AgL-2DDA]. Diffractogram presents data converted for copper–K-α source

Figure 12 presents the typical diffraction peaks from Ag crystal structure. Observed by powder XRD diffraction peaks at 2θ = 38.65, 43.62, 64.70, and 77.20° can be indexed to the (111), (200), (220), and (311) planes of face-centered cubic lattice of metallic silver, respectively (Kashiwagi et al. 2006). The main source of the line broadening of the powder specimen XRD peaks arises from the small particle size and the presence of even smaller crystallites inside the nanoparticles. A specimen broadening can be further widened by inhomogeneous strain and/or defects in single crystalline domains. The mean size of crystallites calculated from the Williamson-Hall plot obtained from the diffraction pattern was 1.9 nm, i.e., smaller than that obtained from the SEM image, as expected.

**Fig. 13:**
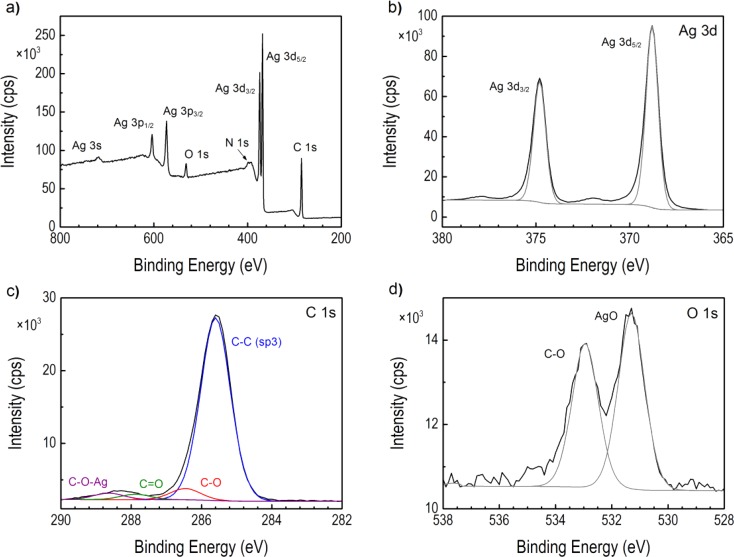
**a** Widescan XPS spectrum of Ag NPs sample. **b** High-resolution scans of Ag3d and **c** C1s and **d** O1s states for Ag NPs capped by laureate/DDA

XPS analysis enabled further insight into the composition of the surface of the Ag NPs. The XPS spectrum and the high-resolution XPS window of the core level atoms comprising the Ag nanoparticles capped with carboxylate/DDA are presented in Fig. 13. The surface scan spectra showed the presence of Ag, C, O, and N atoms according to their binding energies. The most prominent signal in the XPS spectrum is the Ag 3s consisting of two spin-orbit components at 368.8 (Ag3d_5/2_) and 374.8 (Ag3d_3/2_) eV and separated by 6.0 eV (Fig. 13b). Moreover, the deconvolution of Ag (3d) doublet revealed asymmetric peak shape. These two characteristics indicate that the Ag exists in the metallic form. Energy loss features at 371.9 and 378.0 eV are observed at the higher binding energy side of each spin-orbit component for Ag3d metal. These results are in good agreement with the XRD characterization. XPS high-resolution scan for the C1s core level (Fig. 13c) showed the presence of four different peaks. The main peak centered at 285.56 eV was attributed to C–C (sp3), while the peaks at 286.3, 288.0, and 288.8 eV were attributed to C–O, C═O, and C–O–Ag, respectively, in the Ag NPs structure. The doublet for O1s at 531.97 and 530.33 eV was assigned to metal carbonates and AgO species, respectively. Therefore, XPS studies supported results obtained from IR studies on coordination mode of carboxylate moiety.

## Conclusions

(bis)Alkylamine silver carboxylate complexes were synthesized via ligand insertion to verify a mechanism of high-temperature synthesis of silver nanoparticles. The complexes are easily formed even at ambient conditions in nonpolar solvents from suspension of silver carboxylate and dissolved amine. Thermal characterization using DSC and TGA allowed estimating its thermal stability and kinetics of decomposition. Either in solution or in the solid state, amine ligands in the silver carboxylate complexes are *trans*-coordinated. At solid state, silver carboxylates are forming head-to-head dimer configuration, while interacting with primary aliphatic amines they change the coordination character from the bridging bidentate (*Δν* = 103 cm^−1^) to chelating bidentate (*Δν* = 159 cm^−1^). This was deduced from the IR frequency separation, *Δν*, between asymmetrical and symmetrical stretching band positions of silver carboxylate-(bis)amine adduct as compared to similar structures of the corresponding silver and sodium carboxylate and carboxylic acid/amine ion pair. The chelating mode of coordination may result from symmetrical interactions of amine molecules with silver cations. Powder XRD analysis and solid-state ^13^C NMR study have shown layered structures of (bis)amine-silver carboxylate complexes. For example, in the case of [AgOAc-2DDA] complex, the layers are separated by ~30 Å and suggest chain interdigitation of aliphatic amines.

Solution NMR studies allowed to characterize the formed amine/silver carboxylate adduct and therefore to better understand the surface chemistry of Ag NPs stabilized by carboxylates. Coordination of the amine to the metal center of the silver carboxylate was confirmed by solution ^1^H, ^13^C NMR, and diffusion-NMR spectroscopy. The most inner −C_α_H_2_− protons and carbons of carboxylate and amine moieties are deshielded with respect to pure carboxylic and amine ligands. Self-diffusion coefficients, measured by PFG NMR spectroscopy, for OA alone (*D* = 17.9 × 10^−10^ m^2^ s^−1^) and OctAc (*D* = 11.4 × 10^−10^ m^2^ s^−1^) were significantly higher than those measured for the complex (bis)octylamine silver octanoate for which *D* values were respectively *D* = 7.2 × 10^−10^ m^2^ s^−1^ and *D* = 3.9 × 10^−10^ m^2^ s^−1^. Such observations were indicative of a fast exchange on the NMR timescale between free and coordinated amine molecules.

Using [AgL-2DDA] precursor and high-temperature decomposition method, highly monodispersed metallic NPs with spherical morphology were fabricated. The role of primary amine ligands in the synthesis of silver nanoparticles is to lower the decomposition temperature of silver carboxylate. Amine does not directly take part in the reduction mechanism of silver anion. The same reduction mechanism as for silver carboxylate is applied (Eq. 2) provided that OOC–C bond breaking starts at mild conditions shifted by ~60 °C. After the decomposition process, amine with carboxylic acid can form either amide or an ion pair. The average size of Ag NPs was found to be ~5 nm. It turned out that the purified Ag NPs were stabilized mainly by the carboxylate moiety. The IR results indicated that the dodecanoic acid is chemisorbed as a carboxylate onto the Ag nanoparticle surface, and that both oxygen atoms in the carboxylate are coordinated symmetrically to the Ag^(0)^ atoms. This bidendate coordination mode is evidenced by the observation of an O1s peak at 530.33 eV in the XPS spectrum. Amine coordination to the nanoparticle surface is prevented by the presence of a sterically coordinated carboxylate to silver cations. Ag NPs were further characterized using UV–Visible and XRD methods, which show the typical characteristics for silver particles obtained without primary amine in the medium.

The scheme of complex formation examined here can be extended for complexes of amines with other metal carboxylates.
